# Improving the use of research evidence in guideline development: 3. Group composition and consultation process

**DOI:** 10.1186/1478-4505-4-15

**Published:** 2006-11-29

**Authors:** Atle Fretheim, Holger J Schünemann, Andrew D Oxman

**Affiliations:** 1 Norwegian Knowledge Centre for the Health Services, P.O. Box 7004, St. Olavs plass, N-0130 Oslo, Norway; 2 INFORMA, S.C. Epidemiologia, Istitituto Regina Elena, Via Elio Chianesi 53, 00144 Rome, Italy

## Abstract

**Background:**

The World Health Organization (WHO), like many other organisations around the world, has recognised the need to use more rigorous processes to ensure that health care recommendations are informed by the best available research evidence. This is the third of a series of 16 reviews that have been prepared as background for advice from the WHO Advisory Committee on Health Research to WHO on how to achieve this.

**Objective:**

In this review we address the composition of guideline development groups and consultation processes during guideline development.

**Methods:**

We searched PubMed and three databases of methodological studies for existing systematic reviews and relevant methodological research. We did not conduct systematic reviews ourselves. Our conclusions are based on the available evidence, consideration of what WHO and other organisations are doing and logical arguments.

**Key questions and answers:**

What should be the composition of a WHO-panel that is set up to develop recommendations?

The existing empirical evidence suggests that panel composition has an impact on the content of the recommendations that are made. There is limited research evidence to guide the exact composition of a panel. Based on logical arguments and the experience of other organisations we recommend the following:

• Groups that develop guidelines or recommendations should be broadly composed and include important stakeholders such as consumers, health professionals that work within the relevant area, and managers or policy makers.

• Groups should include or have access to individuals with the necessary technical skills, including information retrieval, systematic reviewing, health economics, group facilitation, project management, writing and editing.

• Groups should include or have access to content experts.

• To work well a group needs an effective leader, capable of guiding the group in terms of the task and process, and capable of facilitating collaboration and balanced contribution from all of the group members.

• Because many group members will not be familiar with the methods and processes that are used in developing recommendations, groups should be offered training and support to help ensure understanding and facilitate active participation.

What groups should be consulted when a panel is being set up?

We did not identify methodological research that addressed this question, but based on logical arguments and the experience of other organisations we recommend that as many relevant stakeholder groups as practical should be consulted to identify suitable candidates with an appropriate mix of perspectives, technical skills and expertise, as well as to obtain a balanced representation with respect to regions and gender.

What methods should WHO use to ensure appropriate consultations?

We did not find any references that addressed issues related to this question. Based on logical arguments and the experience of other organisations we believe that consultations may be desirable at several stages in the process of developing guidelines or recommendations, including:

• Identifying and setting priorities for guidelines and recommendations

• Commenting on the scope of the guidelines or recommendations

• Commenting on the evidence that is used to inform guidelines or recommendations

• Commenting on drafts of the guidelines or recommendations

• Commenting on plans for disseminating and supporting the adaptation and implementation of the guidelines or recommendations.

• Key stakeholder organisations should be contacted directly whenever possible.

• Consultation processes should be transparent and should encourage feedback from interested parties.

## Background

The World Health Organization (WHO), like many other organisations around the world, has recognised the need to use more rigorous processes to ensure that health care recommendations are informed by the best available research evidence. This is the third of a series of 16 reviews that have been prepared as background for advice from the WHO Advisory Committee on Health Research to WHO on how to achieve this.

Health care recommendations that are systematically and transparently developed and well informed by the best available evidence require several types of evidence and judgements. Judgements must be made about:

• The expected effects of the options that are being considered,

• Factors that might modify the expected effects in specific settings,

• Needs, risks and resources in specific settings,

• Ethical, legal and political constraints, and

• The balance between the expected benefits harms and costs if a recommendation is implemented.

A group developing recommendations must be capable of assessing the evidence that is available to inform these judgements and to make all of these different types of judgements. In this paper we address the following questions:

• What should be the composition of a WHO-panel that is set up to develop recommendations?

• What groups should be consulted when a panel is being set up?

• What methods should WHO use to ensure appropriate consultations?

Questions related to group processes or integrating values and consumer involvement are addressed in two other papers in this series [1,2].

## What WHO is doing now

Expert committees are sometimes used by WHO to provide guidance. The Director General selects committee members from WHO's expert advisory panels. The members of these panels are primarily included based on "their technical ability and experience". When an expert committee is assembled it should have: "equitable geographical representation, gender balance, a balance of experts from developed and developing countries, representation of different trends of thought, approached and practical experience in various parts of the world, and an appropriate interdisciplinary balance" [3].

Establishing an expert committee is a formal process with regulations established by the World Health Assembly. Many WHO recommendations are not developed by expert committees. Less formal procedures that are not subject to the Regulations for Expert Advisory Panels and Committees are frequently used to convene groups that develop guidelines or recommendations. "Consultations" or "proceedings" also frequently provide the basis for recommendations. Consumers or representatives of the general public are rarely included in groups that develop recommendations.

The Guidelines for WHO Guidelines state that the "Technical Guidelines Development group" should be multidisciplinary with around 8–12 individuals representing stakeholders (professionals, disease experts, primary care/public health, end users, and patients) as well as methodologists [4]. However, up to now WHO has published few recommendations that have adhered to these guidelines.

Broad consultations do not appear to be commonly used to identify potential members of expert committees or other groups that develop guidelines or recommendations. Consultations at other stages during the process of developing recommendations are also uncommon, apart from peer review of draft reports. Occasionally, draft recommendations may be circulated more widely.

## What other organisations are doing

In a recent international survey of 152 units that support the use of research in developing guidelines and health policy, most respondents reported that their guidelines development panels consisted of several stakeholders or expert groups – often including end-users and consumers [5]. Another review of guidelines on hypertension and hyperlipidaemia, found that stakeholder involvement was much lower in guidelines sponsored by specialty societies than in guidelines sponsored by other groups [6]. A third review of 18 prominent guideline organisations in Australia, Canada, Europe, New Zealand and the U.S. found that guideline development groups typically consist of 10 to 20 members and the number of disciplines per group is often three to five [7]. Most of these programs invite methodological experts (epidemiologists, library scientists and others) and patient representatives. Most have permanent staff providing editorial support.

Many agencies have issued guidelines for developing guidelines [8-11] (see also our review of Guidelines for Guidelines [12]), and all such documents that we are aware of recommend convening multi-disciplinary groups. This includes consumers, professionals working in the field, and individuals with the necessary methodological skills (e.g. epidemiologists, economists). Procedures for recruitment of panel members are usually prescribed or suggested, with emphasis on extensive consultation with relevant stakeholder-groups.

In contrast to approaches that rely heavily on clinical experts or research experts, as exemplified by many specialty societies [6] and the WHO, the National Institute for Health and Clinical Excellence (NICE) in the UK does not necessarily include experts in their guideline development groups (GDGs): "Experts attending a GDG are present because of their knowledge in a particular area. Therefore, it is important that they sit within the group and enter fully into any discussion. However, they are not full members of the group; they do not have voting rights and should not be involved in the final wording of recommendations." The role of the professional members in the GDG is to "represent the perspective(s) of the health care workers involved" [10]. In an external evaluation carried out by WHO, it was concluded that NICE is "internationally a leading agency", and that the organisation "has developed a well-deserved reputation for innovation and methodological development" [13].

Most guidelines for guidelines highlight the importance of having an effective leader. This person has a key role in facilitating "the interpersonal aspects of the group processes" and ensuring "that the group works in a spirit of collaboration, with a balanced contribution from all members" [10].

Some agencies arrange training for members of GDGs – particularly, but not solely, aimed at facilitating the active participation of consumer representatives [14]. Another suggested approach to ensure consumer involvement is to establish separate focus groups for this purpose [10].

Wide consultation in the course of developing recommendations may be done in various ways, for instance by hosting open meetings to discuss guideline drafts [8], or by posting guideline drafts on the web [10]. Peer-review is also commonly used. In the survey of 152 units that support the use of research in development of guidelines and health policy, most reported involvement of target-users in the selection of topics, e.g. in priority-setting groups, through surveys or by reviewing draft lists of priority topics [5]. Most respondents also reported having consumers involved at various stages of the development process, often by review of draft guidelines or reports [5]. In its manual for guideline developers, NICE specifies several stages during guideline development where consultations with stakeholders should take place:

• When the draft scope of the guideline has been prepared

• During the selection of panel members

• When the full draft version of the guidelines is completed

## Methods

The methods used to prepare this review are described in the introduction to this series [15]. Briefly, the key questions addressed in this paper were vetted amongst the authors and the ACHR Subcommittee on the Use of Research Evidence (SURE). We searched PubMed and three databases of methodological literature (the Cochrane Methodology Register [16], the US National Guideline Clearinghouse [17], and the Guidelines International Network [18]) for existing systematic reviews and relevant methodological research that address these questions. We did not conduct systematic reviews ourselves. The answers to the questions are our conclusions based on the available evidence, consideration of what WHO and other organisations are doing, and logical arguments.

In our literature search on panel composition we used the terms "group composition" or "panel composition" or "'consumer involvement' and guidelines". We also checked the reference lists of key papers and contacted key researchers in the field. In our search on consultation processes, we used the term "guidelines and consultation and process".

## Findings

### What should be the composition of a WHO-panel that is set up to develop recommendations?

We identified relatively few articles on group composition. A key paper was a comprehensive report by Murphy and colleagues from 1998, who reviewed the research literature on group composition and clinical guideline development [19]. This systematic review identified several studies that compared recommendations by groups with different compositions, and several comparisons of judgements made by homogenous subgroups of mixed groups [19]. The authors found that "these studies, although few in number, show that differences in group composition may lead to different judgements. More specifically, members of a specialty are more likely to advocate techniques that involve their specialty." Their conclusion was that "these studies confirm that the composition of groups is important in determining the decision reached."

Knowing that groups with different compositions produce different recommendations does not necessarily tell us what group composition will provide the most appropriate recommendations. Arguments for using multidisciplinary groups are largely based on logic. For example, "Individuals' biases may be better balanced in multidisciplinary groups, and such balance may produce more valid guidelines" [20]. A report from the U.S. Institute of Medicine (IOM) put forward three arguments for multidisciplinary groups: 1) Multidisciplinary participation increases the probability that all relevant scientific evidence will be located and critically evaluated; 2) Such participation increases the likelihood that practical problems with using guidelines will be identified and addressed; 3) Participation helps build a sense of involvement or "ownership" among different audiences for the guidelines [21].

Arguments against having narrowly focused expert groups are based in part on research that have compared expert recommendations to systematic reviews and which have investigated the relationship between expertise and systematic reviews. In one study comparisons were made between recommendations of clinical experts in textbooks and major medical journals and results of meta-analyses of randomized controlled trials of treatments for myocardial infarction [22]. The investigators found that clinical experts often made recommendations that were not consistent with available research findings. Another study found strong correlations between the expertise of authors of reviews and the methods that were used in the reviews [23]. Expertise was associated with stronger opinions prior to conducting a review, less time spent conducting a review, and the use of less systematic and transparent methods.

The systematic review by Murphy and colleagues included studies of the effects that heterogeneity has on group judgement, and concluded that "The weight of evidence suggest that heterogeneity in a decision-making group can lead to a better performance than homogeneity. There is, however, also some evidence that heterogeneity may have an adverse effect because conflict may arise between diverse participants" [19].

In addition, the review found few studies of the extent to which the particular individuals that participate in a group affect the groups' decisions. The authors concluded that "the selection of individuals has some, but not a great deal, of influence on outcome", based on this limited research.

Finally, the review included research related to the optimal size of groups. The authors remark that "having more group members will increase the reliability of group judgement", while "large groups may cause coordination problems". They base their conclusion mainly on research within social and organisational psychology: "It is likely that below about six participants, reliability will decline quite rapidly, while above about 12, improvements in reliability will be subject to diminishing returns."

We have identified one recent study not included in the review by Murphy et al. where recommendations made by groups of different composition were compared [24], as well as four studies comparing judgements made by different subgroups in mixed groups [25-28]. The findings, which are consistent with the conclusions of the review, indicate that clinical experts have a lower threshold for rating the procedures they perform as being appropriate. Another study, in which clinicians were surveyed about the appropriateness of coronary angiography for various indications, found a similar relationship [29].

We did not find any studies on the impact of group composition on public health or health systems recommendations. However, there is some evidence that suggests that the same relationships between expertise and recommendations that have been found for clinical recommendations are also found for public health recommendations; i.e. that expert recommendations are frequently not consistent with the available research evidence [30]. Moreover, those making public health and health policy recommendations may frequently not systematically consider potential adverse effects of public health and health policy interventions.

We found several systematic reviews of consumer involvement [31-35]. There are a number of relevant arguments for including consumers in groups that develop recommendations and descriptions of practical experience. However, a review of comparative studies of interventions to promote consumer involvement [31], found a "lack of evidence from comparative studies to inform decisions about desirable and adverse effects of consumer involvement in collective decisions about health care or how to achieve effective consumer involvement". The authors of another systematic review, which included non-comparative studies of how to involve consumers in setting the research and development agenda for health systems [32], recommend collaborating with "well-networked consumers and providing them with information, resources and support to empower them in key roles for consulting with their peers." They also recommended "consultations should engage consumer groups directly and repeatedly in facilitated debate." See also our review on how to integrate values and involve consumers in guideline development [2].

### What groups should be consulted when a panel is being set up?

We did not identify papers addressing this question.

### What methods should WHO use to ensure appropriate consultations?

We did not identify any research findings that could inform the answer to this question

## Discussion

Based on the findings from the reports we have identified, there is sufficient evidence to conclude that how a panel is composed can have an important impact on conclusions drawn by a group when making recommendations for health care. In particular, clinical experts are more likely to recommend procedures linked to their own specialty than others. Furthermore, experts in a field frequently do not employ systematic methods when reviewing evidence and developing recommendations. These findings support the current recommendation in the Guidelines for WHO Guidelines: Panels should be multidisciplinary, including a broad representation of stakeholders, as well as methodologists [4].

The research evidence to guide panel composition and consultation processes is limited. However logical arguments and the experience of other organisations suggest that

• Groups that develop guidelines or recommendations should be broadly composed and include important stakeholders such as consumers, health professionals that work within the relevant area, and managers or policy makers.

• Special attention should be paid to the selection of a group leader who has a crucial role in ensuring a positive group process and that all voices within the group can be heard.

• Wide consultations should be done when selecting members of a group to develop WHO recommendations, for example by direct contact with stakeholder groups.

• Groups should include or have access to individuals with the necessary technical skills, including information retrieval, systematic reviewing, health economics, group facilitation, project management, writing and editing.

• Groups should include or have access to content experts.

• Many group members will not be familiar with the methods and processes that are used in developing recommendations, and should be offered training and support to help ensure understanding and facilitate active participation.

The process of developing recommendations, including the selection of group members, should be transparent. The process should also include wide consultation that encourages feedback at subsequent steps in the process, which may include:

• Identifying and setting priorities for guidelines and recommendations

• Commenting on the scope of the guidelines or recommendations

• Commenting on the evidence that is used to inform guidelines or recommendations

• Commenting on drafts of the guidelines or recommendations

• Commenting on plans for disseminating and supporting the adaptation and implementation of the guidelines or recommendations.

• Commenting on what research should be conducted based on the guidelines

## Further work

We have not conducted an exhaustive systematic review, but have based much of this paper on a systematic review from 1998. We have not found subsequent studies that provide conflicting evidence. There is, however, limited research for the questions addressed in this report. We do not believe that a more exhaustive review would yield a great deal of additional evidence at this time. However, it would be valuable for WHO or others to undertake and keep up-to-date systematic methodology reviews that address specific issues of group composition, including the selection of a group leader, methods for effective consultations, and methods for effective consumer involvement.

## Competing interests

AF and ADO work for the Norwegian Knowledge Centre forthe Health Services, an agency funded by the Norwegian government that produces systematic reviews and health technology assessments. All three authors are contributors to the Cochrane Collaboration. ADO and HJS are members of the GRADE Working Group. HJS is documents editor and chair of the documents development and implementation committee for the American Thoracic Society and senior editor of the American College of Chest Physicians' Antithrombotic and Thrombolytic Therapy Guidelines.

## Authors' contributions

AF prepared the first draft of this review. HJS and ADO contributed to drafting and revising it.
